# Supplementary material for: Sodium Hyaluronate Nanocomposite Respirable Microparticles to Tackle Antibiotic Resistance with Potential Application in Treatment of Mycobacterial Pulmonary Infections
Source: Pharmaceutics. 2019 May 1;11(5):203. doi: 10.3390/pharmaceutics11050203 (PMC6571635; doi:10.3390/pharmaceutics11050203)
Supplement: Supplementary file 1 [file pharmaceutics-11-00203-s001.pdf]

Supplementary Materials: Sodium Hyaluronate Nanocomposite Respirable Microparticles to Tackle Antibiotic Resistance with Potential Application in Treatment of Mycobacterial Pulmonary Infections

Irene Rossi [1,2], Francesca Buttini [1,2], Fabio Sonvico [1,2], Filippo Affaticati [1], Francesco Martinelli [1,†], Giannamaria Annunziato [1], Diana Machado [3], Miguel Viveiros [3], Marco Pieroni [1] and Ruggero Bettini [1,2,*]

1. Formulation Design

A preliminary formulation study was carried out to optimize the starting suspension in terms of total solid concentration and components ratio. This study was done employing firstly laser light diffraction (Spraytec®, Malvern, UK) and, successively, dynamic light scattering (Zetasizer nano series ZEN3600, Malvern, UK) to determine the size of the HA particles obtained with the ethanol antisolvent effect.

The first suspension (1) was prepared starting from a water (30% *v*/*v*) solution of HA low molecular weight (0.83% *w*/*v*), based on the solution/suspension equilibrium plot (Figure 2) and what reported by Martinelli et al. [38]. As s first approach, a ratio 1:1:1:1 between all components (HA:isoniazid:rifampicin:verapamil) was adopted. The suspension obtained was composed by several different populations (Span = 24.26) in particle size (Figure S1).

Successively, components ratio was changed based on Chan et al. [39] and Parumasivam et al. [72] publications. A new formulation was produced employing always 0.83% *w*/*v* of HA, a ratio 1:3 between antibiotics (rifampicin+isoniazid) and verapamil, and a ratio 1:2 between isoniazid and rifampicin. The total amount of rifampicin was the limiting one, after the determination of its solubility in the ethanol fraction (70% *v*/*v*), was used a concentration 4 mg/mL. The monomodal cumulative curve of the particle size of this second formulation, after 30 minutes of Ultra-TURRAX®, is reported in Figure S1.

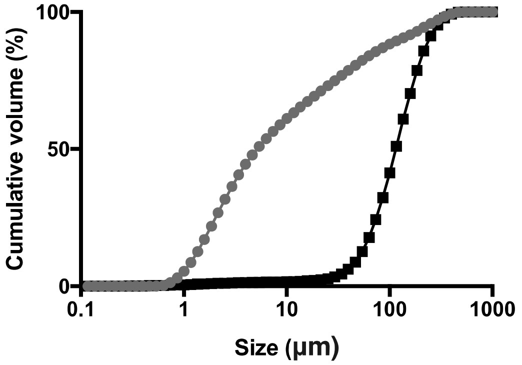

Figure S1. Particle size cumulative undersize distribution evaluated by laser light diffraction of suspension 1 (circle) and of suspension 2 (square).

Because the $D_{v,50}$ of the second formulation was around 100 µm, in the third suspension tested the HA concentration was reduced to 0.2% *w*/*v*, maintaining the same ratios between the other components. After 30 minutes of Ultra-TURRAX®, the suspension was analyzed by DLS (mean diameter 405.1 ± 5.35 nm), because it was not possible to obtain a size distribution using Spraytec®. The ratios of this last nanosuspension were the same of the nanosuspension spray dried, with a total solid concentration of 0.59 *w*/*v*.

Table S1. Mobile phase gradient and flow as well as wavelengths of the UV detector used to quantify the three drugs by HPLC: potassium phosphate buffer (A), methanol (B), ultrapure water (C) and acetonitrile (D).

Time (min)	A (%)	B (%)	C (%)	D (%)	Flow rate (mL/min)	Wavelength (nm)
0–7	90	10			1.0	261
7–8		5	95			
8–10			90	10	1.2	
10–12			80	20		278
12–34			63	37	1.5	
34–35	60			40	1.2	
35–52	50			50	1.0	254
52–55	90	10				261

2. Differential Scanning Calorimetry Analysis of the Spray Dried Powder

DSC trace of the spray dried powder (Figure S2) presented a broad endothermic event between 25 and 130 °C, reasonably due to the evaporation of residual water bonded to the polymer. This phenomenon was followed by an exothermic peak at high temperature (210–285 °C) ascribable to the thermal degradation of one or more components of the powder (likely HA).

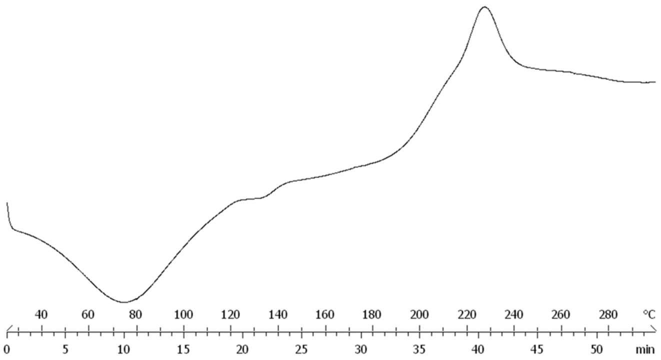

Figure S2. DSC thermograms of the spray dried powder.

3. Solid State of the Spray Dried Powder

The solid state of the spray dried powder was analyzed by PXRD. Figure C reported the X-ray intensity in counts per second (cps) observed for each angle 2θ. From the diffraction pattern is clear that the crystalline nature of the three drugs was completely lost after the spray drying process.

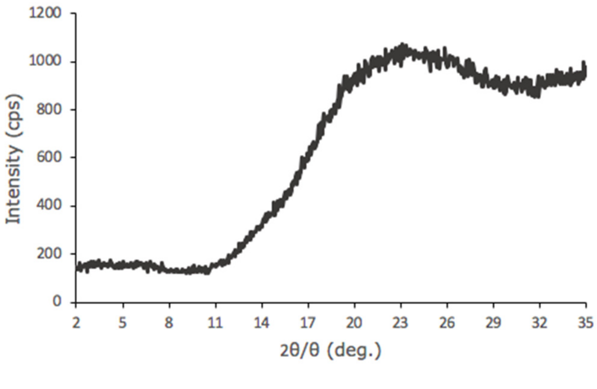

Figure S3. X-ray plot of the spray dried powder.

References

38. Karimi, K.; Katona, G.; Csóka, I.; Ambrus, R. Physicochemical stability and aerosolization performance of dry powder inhalation system containing ciprofloxacin hydrochloride. *J. Pharm. Biomed. Anal.* **2018**, *148*, 73–79.
39. Caviedes, L.; Delgado, J.; Gilman, R.H. Tetrazolium Microplate Assay as a Rapid and Inexpensive Colorimetric Method for Determination of Antibiotic Susceptibility of Mycobacterium tuberculosis. *J. Clin. Microbiol.* **2002**, *40*, 1873–1874.
72. Makino, K.; Nakajima, T.; Shikamura, M.; Ito, F.; Ando, S.; Kochi, C.; Inagawa, H.; Soma, G.-I.; Terada, H. Efficient intracellular delivery of rifampicin to alveolar macrophages using rifampicin-loaded PLGA microspheres: Effects of molecular weight and composition of PLGA on release of rifampicin. *Colloids Surf. B* **2004**, *36*, 35–42.

© 2019 by the authors. Submitted for possible open access publication under the terms and conditions of the Creative Commons Attribution (CC BY) license (http://creativecommons.org/licenses/by/4.0/).
